# PK/PD Analysis of High-Dose Daptomycin Use in the Treatment of Bone and Joint Infections: Data from a Real-World Setting

**DOI:** 10.3390/microorganisms13020304

**Published:** 2025-01-30

**Authors:** Jacopo Angelini, Simone Giuliano, Francesco Russiani, Francesco Lo Re, Sarah Flammini, Barbara Cadeo, Luca Martini, Carlo Tascini, Massimo Baraldo

**Affiliations:** 1 Clinical Pharmacology and Toxicology Institute, University Hospital Friuli Centrale ASU FC, 33100 Udine, Italy; jacopo.angelini@asufc.sanita.fvg.it (J.A.); francesco.russiani@asufc.sanita.fvg.it (F.R.); francesco.lore@asufc.sanita.fvg.it (F.L.R.); massimo.baraldo@uniud.it (M.B.); 2 Department of Medicine (DMED), University of Udine (UNIUD), 33100 Udine, Italy; carlo.tascini@asufc.sanita.fvg.it; 3 Infectious Diseases Clinic, Department of Medicine (DMED), University of Udine and Azienda Sanitaria Universitaria Friuli Centrale, 33100 Udine, Italy; sarah.flammini@asufc.sanita.fvg.it (S.F.); barbara.cadeo@asufc.sanita.fvg.it (B.C.); lucamartini9@gmail.com (L.M.); 4 Department of Medicine, Surgery and Health Sciences, University of Trieste, 34149 Trieste, Italy

**Keywords:** daptomycin, therapeutic drug monitoring, pharmacokinetics, pharmacodynamic, bone and joint infections, infectious diseases

## Abstract

Background: Daptomycin is widely used in bone and joint infections (BJIs) caused by Gram-positive cocci. The pharmacokinetics of daptomycin are characterized by relevant variability in terms of drug exposure. Due to these pharmacological properties, the dosing suggested by the Summary of medical Product Characteristics could result in sub-therapeutic or toxic concentrations, especially considering the high doses recommended for BJIs. Therapeutic Drug Monitoring (TDM) of daptomycin helps clinicians in verifying the patient’s exposure, due to the lack of pharmacokinetic/pharmacodynamic (PK/PD) data in this clinical setting. Methods: We retrospectively analyzed 170 daptomycin plasma concentrations of 77 patients with BJIs from July 2022 to December 2023. We focused on the pharmacokinetics of daptomycin to investigate when drug plasma concentrations achieved adequate PK/PD targets. Results: In the first TDM, 7.8% of patients were underexposed according to the estimated area under the curve (eAUC_0–24h_ < 666 mg·h/L), whereas 35.1% were on target according to both the eAUC and trough plasma concentration (eAUC_0–24h_ 666 − 939 mg·h/L; C_min_ < 24.3 mg/L). The patients who were overexposed had trough plasma concentrations > 24.3 mg/L (27.3%) or eAUC_0–24h_ > 1174 mg·h/L (33.8%). Differences in drug exposure were observed according to weight and sex. Conclusions: Due to the difficult management of this drug’s dosing, analyzing daptomycin plasma concentrations through TDM represents a powerful tool in BJIs.

## 1. Introduction

Bone and joint infections (BJIs) represent difficult-to-treat infections that clinicians must manage, and they include Prosthetic Joint Infections (PJIs), osteomyelitis and infective arthritis. Considering PJIs alone, they represent a significant and increasing cause of morbidity. The cumulative incidence of PJIs is approximately 1–2% over the lifetime of the prosthetic joint [1]. However, the absolute number of infections is expected to rise due to the substantial increase in total joint arthroplasty (TJA) procedures. Indeed, between 2000 and 2014, the volume of total hip arthroplasty (THA) increased by 132%, from 159,856 to 370,770 procedures [2]. Similarly, the volume of total knee arthroplasty (TKA) rose by 148%, from 274,025 to 680,150 procedures during the same period [2]. Moreover, globally, the volume of total hip arthroplasty is projected to further double by 2030 [2].

These conditions usually result from infectious complications of surgical procedures involving the joints, hematogenous spread, or the contiguous extension of pre-existing infectious processes in other anatomical areas or those associated with internal fixation. Regardless of the cause, these clinical scenarios require prompt and effective therapeutic intervention to resolve the infection. Appropriate antibacterial therapy, combined with surgical source control, plays a crucial role in achieving this. Source control is particularly crucial in revision procedures for PJIs, as preventing contamination of bone cements with bodily fluids (e.g., blood, synovial fluid, and fat) and 0.9% NaCl solution during surgery is essential [3]. Moreover, the use of antibiotic bone cements (ABCs), including traditional antibiotic-loaded cements (ABLCs) and advanced formulations containing novel antibacterial agents such as N-halamine, represents a key strategy for optimizing surgical outcomes and reducing the risk of infection recurrence [4]. Additionally, admixing bone cement with 2% hydroxyapatite has been shown to significantly improve the compressive strength of the cement, while also contributing to a reduction in infection rates, further enhancing its effectiveness in the surgical management of PJIs [5]. Alongside surgery, the other cornerstone in the management of BJIs is antibiotic therapy, which plays a critical role in eradicating the infection and preventing infection relapse. Daptomycin is a cyclic lipopeptide that is preferred to many other antibiotics, such as vancomycin, in the treatment of BJIs by Gram-positive cocci due to its various pharmacological properties, which include bactericidal activity against many antibiotic-resistant Gram-positive bacteria [6], effective action within biofilms [7,8], and good bone and synovial penetration [9,10,11]. Focusing on the mechanism of action, daptomycin, by complexing with calcium ions, inserts itself into the bacterial membrane, disrupting its integrity and causing the release of potassium ions, magnesium, and adenosine triphosphate. This leads to rapid membrane depolarization, resulting in the inhibition of protein, DNA, and RNA synthesis. Consequently, bacterial cell death occurs rapidly without inducing immediate cell lysis [12,13]. It is administered via intermittent intravenous infusion, lasting 30 min [14]. However, daptomycin exhibits a safety profile primarily characterized by two serious side effects typically associated with this drug: daptomycin-induced eosinophilic pneumonia (DIEP) and muscle toxicity [14,15].

Although the drug has been on the market for a long time and is widely used, these adverse events have not yet been associated with specific and conclusive risk factors that could serve as indicators of the likelihood of daptomycin toxicity. The most robust evidence is related to a high inflammatory burden in the patient and excessive drug exposure [15,16]. Regarding the latter aspect in particular, some pharmacokinetic parameters have been identified that appear to be more strongly associated with a higher risk of toxicity: daptomycin plasma concentrations measured a few minutes before administration, representing its minimum concentration in the dosing (trough plasma drug concentrations; C_min_) > 24.3 mg/L [17], or the overall exposure to the antibiotic in the dosing represented by the area under the concentration–time curve over 24 h (AUC_0–24h_) > 939 mg·h/L [15] or >1174 mg·h/L [18], measured by multiple samples over 24 h or by a specific validated formula published in the literature [19].

Daptomycin is used off-label for the treatment of BJIs by administering high doses (≥8 mg/kg). For this reason, in this clinical condition, relevant concerns arise, looking for a balance between efficacy and safety [20]. On top of this, due to the significant pharmacokinetic variability of this molecule, the Clinical Pharmacological Institute of our University Hospital routinary performs Therapeutic Drug Monitoring (TDM) of numerous antimicrobial treatments to improve their risk profile and maximize their efficacy [21,22,23,24], as reported in position statements of several scientific societies, representing a useful tool against antimicrobial resistance [25,26,27,28,29]. (H) TDM entails the measurement of a prescribed drug in a biological matrix, typically plasma or blood, using single or multiple samples. The resulting data, when appropriately interpreted, directly inform and influence prescribing decisions. Since daptomycin continues to be widely used in various types of infections in cases where the pathogen is sensitive to daptomycin, and considering that the dosing of the drug is primarily dependent on renal function and body weight, TDM for this drug is routinely performed at our center to better tailor therapy with this important pharmacological option [30]. For all the aforementioned reasons, we hypothesize that the use of high-dose daptomycin may represent a clinically significant challenge in managing this drug, with the risk of exposing patients to excessive daptomycin concentrations, even exceeding the PK/PD targets for this type of infection—still insufficiently studied—thereby increasing the risk of dose-dependent side effects associated with this drug. The primary goal of this study is to better characterize daptomycin exposure related to the pertinent pharmacodynamic parameters (PK/PD targets), focusing specifically on BJIs, as there are a lack of data in the literature in this clinical setting and also considering the limited diffusion of units performing TDM due to several challenges that hinder the implementation of a TDM service in clinical practice [31]. The secondary aims of this study are represented by the characterization of the main parameters influencing the exposure of daptomycin and a focus on the efficacy and safety impact of this antibiotic treatment, mainly looking at myotoxicity and DIEP. All these data are interpreted taking in consideration the newest and most relevant studies investigating the use of TDM for daptomycin.

## 2. Materials and Methods

**Figure 1:**
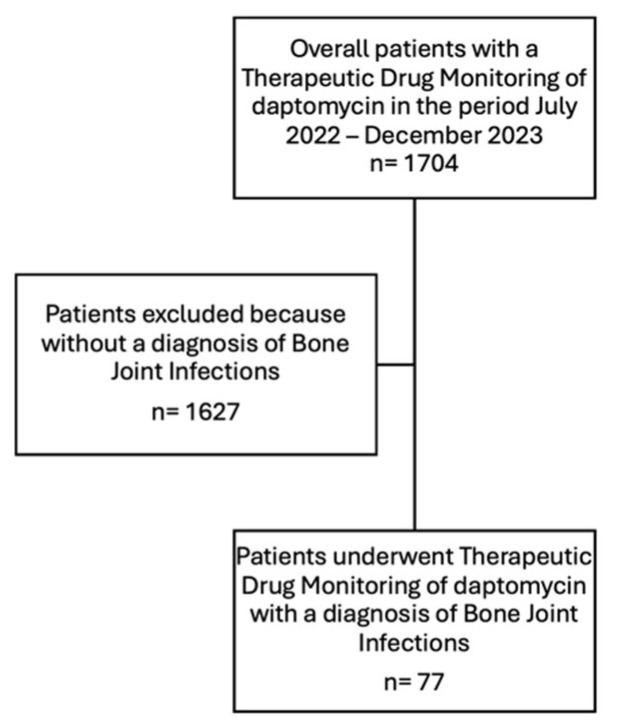
Flowchart of patient inclusion and exclusion criteria for daptomycin PK/PD analysis.

This is a retrospective, monocentric study, including adult patients with a suspected or documented diagnosis of Gram-positive BJI who received daptomycin and whose plasma concentrations were measured once the steady state was reached. The study was approved by the Institutional Review Board of University of Udine (Prot IRB: 266/2023). We included all patients who were hospitalized at our University Hospital from July 2022 to December 2023. Data were retrieved from the hospital’s electronic registry, selecting all TDM performed by the Clinical Pharmacology Institute of ASU FC in the time frame of interest and then identifying the analyses required by the orthopedic wards (see Figure 1).

The employment of daptomycin was at the discretion of the treating physician, who prescribed the starting dose according to clinical conditions, weight and renal function and who could adjust daptomycin dosing according to TDM advice. Pharmacological data, daptomycin dosing and TDM data, clinical conditions, demographic features and laboratory data were retrospectively retrieved from the hospital’s electronic registry. The diagnoses of BJIs were retrieved from the patient-related form clinicians completed when they required the daptomycin TDM. Signed informed consent was waived due to the retrospective nature of this investigation. Patients without main clinical, laboratory or pharmacological parameters such as age, weight, renal function or daptomycin doses or concentrations were excluded from the analyses. Renal clearance was assessed by the Cockroft–Gault formula and then adjusted for the ideal weight. For analyses related to body weight, the lean body weight was also estimated.

Although the role of inflammatory markers in monitoring treatment response has not been clearly defined in the literature, either for osteomyelitis not associated with hardware or for osteomyelitis related to internal fixation devices or PJIs [32], there is, for example, a weak recommendation for monitoring C-reactive protein (CRP) levels between stages of a two-stage revision before reimplantation [1]. Therefore, we attempted to correlate the targeted AUC_0–24h_ with CRP levels measured after treatment. The CRP was obtained at the time of diagnosis and at the end of treatment or between stages in two-stage exchange arthroplasty.

Myotoxicity was assessed by monitoring serum creatinine phosphate kinases (CPKs) and was considered in case of elevation >3 times the upper limit of normal (192 U/L) or elevation of >3 times the baseline concentrations. Eosinophilic counts were evaluated to identify eosinophilia as surrogate markers of DIEP in case of absolute eosinophil count ≥500 eοsinophils/uL [33]. Variation in eosinophilic count was evaluated comparing baseline data to every available follow-up measurement.

All patients performed daptomycin TDM according to the clinical practice, and its daily area under the concentration–time curve (eAUC_0–24h_; including eAUC_24–48h_ in case of other day administration, meaning when the drug dose was given every 48 h instead of every 24 h as indicated in the SmPC as the standard dose) was estimated according to the following Equation (1) reported in the literature [18,19], based on daptomycin trough and peak plasma concentrations:

(1) $${\mathrm{AUC}}_{0–24\;h}\;=\;0.5\;\times \;t^{\prime }\times C_{\max}\;+\int_{t^{\prime }}^{\mathrm{infinity}}C_{\max}\;\times e^{-kel\times (t)}dt-\;\int_{24}^{\mathrm{infinity}}C_{\min}\;\times e^{-kel\times (t)}dt$$

The peak and trough concentrations of daptomycin were determined through blood samples collected 30 min after the drug infusion and before dosing, respectively, on day 3 of therapy, once steady state was achieved. The drug clearance was estimated as apparent clearance, calculated as the ratio between eAUC_0–24_ and the daily dose.

Daptomycin concentrations were measured by a validated High-Performance Liquid Chromatography–Ultraviolet (HPLC-UV) method previously published [34], using Nexera-i 2040 LC, Shimadzu^®^ (Shimadzu USA Manufacturing INC, Canby, OR, USA).

The efficacy and safety considerations derived from the PK/PD analyses are interpreted according to the literature [18,25]. For the analysis of the safety profile of daptomycin, we considered the main PK parameters expressing the overall exposure to the antibiotic and its property to be cleared, such as eAUC_0–24h_ and the trough plasma concentrations, respectively. For the efficacy daptomycin PK/PD targets, we considered the main PK parameter indicating the daily exposure to daptomycin which is represented by the eAUC_0–24h_ and we combined this with the pertinent PD parameter of interest of bacterial disease which is the Minimum Inhibitory Concentration (MIC) of the isolated pathogen. When daptomycin was administered, although the pathogen was not known by the clinicians (empirical treatment), we chose the MIC = 1 mg/L as the PD parameter corresponding to the Epidemiological Cut-Off value (ECOFF) of Staphylococcus aureus according to the European Committee on Antimicrobial Susceptibility Testing (EUCAST) [35].

Statistical analyses were performed with Prism (GraphPad Software version 10.4.0, San Diego, CA, USA). The Shapiro–Wilk normality test was performed for each data set. Continuous variables were described using the median and interquartile range (IQR), and categorical variables were described using counts and frequencies. Principal Component Analysis (PCA) was used to analyze multiple continuous variables, where the two Principal Components (PC1–PC2) are linear combinations of the variables and represent the maximal amount of variance of data. PCA uses covariance matrices to calculate eigenvalues (and the corresponding eigenvectors), and PC1 and PC2 allow one to plot data on a bi-dimensional graph. Each variable is correlated to PC1 and PC2 through the loadings, which vary between −1 and 1, indicating, respectively, strong negative and positive correlations. The p values for the comparison of different subgroups were derived from the Kruskal–Wallis, *t*-test or Mann–Whitney test, according to data distribution, for continuous variables with a post hoc Dunn’s multiple comparison test. Contingency analysis was performed by Fisher’s exact test. Correlation among categorical variables was determined by the chi-square test. Statistical significance was set for a two-tailed *p* value ≤ 0.5.

## 3. Results

### 3.1. Baseline Population Features

This retrospective study included 170 TDM measurements (mean 2 TDMs per patient) from 77 patients, whose features are reported in Table 1. The next TDM scheduling, and consequently the number of TDMs per patient, varied according to parameters such as clinical and laboratory conditions, achievement of PK/PD target, and disease progression. The first TDM for every patient was measured after the steady state for daptomycin was reached. A number of 21 patients underwent only 1 TDM, while 56 patients had multiple measurements. The median interval between measurements was 6 days.

All patients were treated with daptomycin for different conditions: 63 Prosthetic Joint Infections (PJIs), 10 septic arthritis cases and 4 osteomyelitis cases.

### 3.2. Primary Aim: Pharmacokinetics/Pharmacodynamics of Daptomycin in BJIs

Considering all 170 TDM measurements, daptomycin median plasma peak and trough concentrations were, respectively, 81.68 and 17.79 mg/L. The median estimated AUC_0–24h_ was 996.3 mg·h/L (IQR: 836.7–1218 mg·h/L), with a minimum value of 395.7 mg·h/L and a maximum one of 2263 mg·h/L. The median estimated drug clearance was 12.2 mL/min (IQR: 9.4–15.2 mL/min).

When daptomycin doses were administered according to the summary of the product characteristics (SmPC) [14], on first TDM measurement, 6 patients out of 77 (7.8%) were underexposed (eAUC_0–24h_ < 666 mg·h/L), 27 patients (35.1%) showed an eAUC_0–24h_ on target between 666 mg·h/L and 939 mg·h/L with trough concentrations under the toxic threshold of daptomycin (24.3 mg/L), and 16 patients (20.8%) had a high eAUC_0–24h_ to monitor between 939 mg·h/L and 1174 mg·h/L and trough concentrations <24.3 mg/L. Overexposure was documented in 47 patients (61.0%): 21 patients had trough concentrations >24.3 mg/L (27.3%), whereas 26 patients (33.8%) had an eAUC_0–24h_ > 1174 mg·h/L.

**Figure 2:**
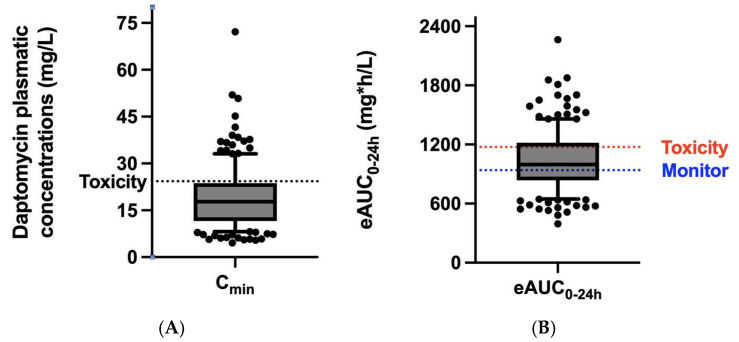
Pharmacokinetic safety targets, indicated by trough (C_min_) plasma concentration of daptomycin (**A**) or by estimated area under the daily concentration–time curve (eAUC_0_–_24h_) (**B**), according to the most up-to-date literature (see references in the main text).

**Figure 3:**
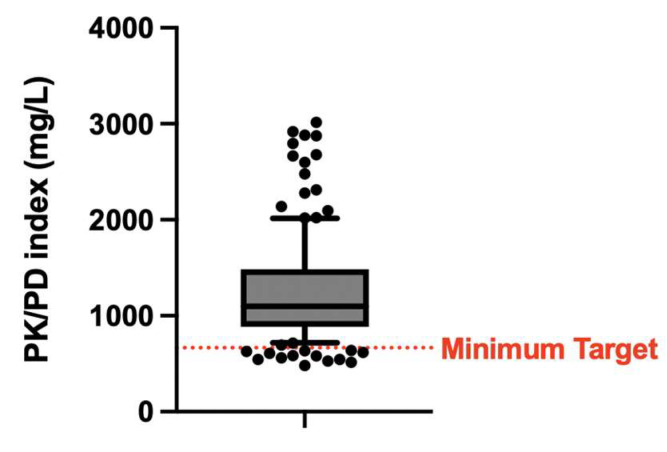
Pharmacokinetic and pharmacodynamic index, considering the plasma concentrations of daptomycin and the pertinent Minimum Inhibitory Concentration, according to the most up-to-date literature (see references in the main text).

The PK targets of safety and efficacy are represented in Figure 2 and Figure 3, respectively.

**Figure 4:**
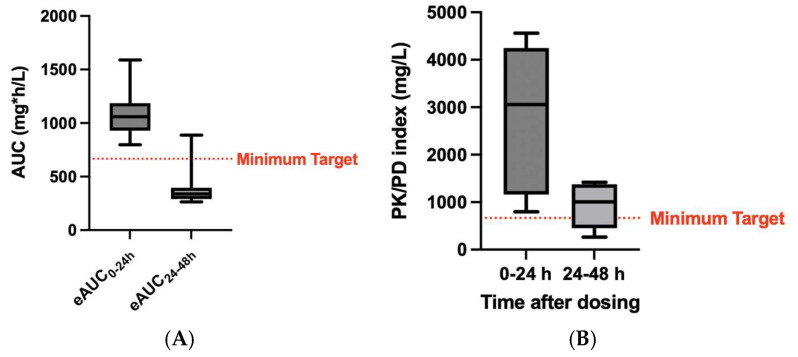
Estimated area under the daily concentration–time curve in different day interval dosing of daptomycin (eAUC_0–24h_ and eAUC_24–48h_) (**A**) and pertinent PK/PD index (**B**).

The estimated AUCs from eight daptomycin TDM results derived from dosing based on a other day administration schedule and the median daily exposure within 24 h from dosing and between 24 and 48 h after dosing were 1060 mg·h/L (IQR: 929.7–1186 mg·h/L) and 339.8 mg·h/L (IQR: 290.5–396.6 mg·h/L), respectively. These second concentrations are far from the minimum daily AUC target, although the pertinent low MICs allow us to obtain the PK/PD target (Figure 4).

### 3.3. Secondary Aims

#### 3.3.1. Patients’ Parameters Influencing Daptomycin Pharmacokinetics

**Figure 5:**
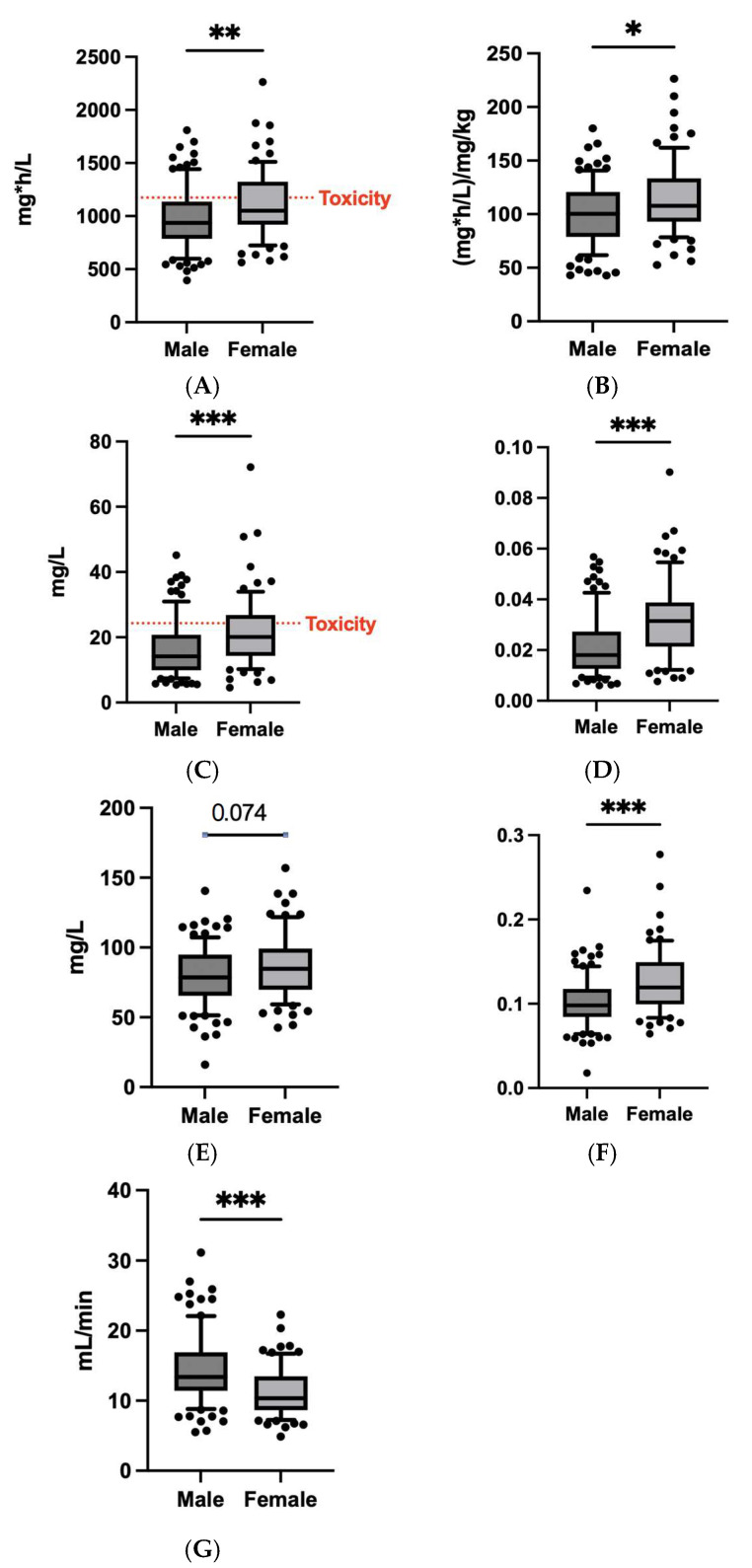
(**A**) Comparison of estimated area under the daily concentration–time curve of daptomycin (eAUC_0–24h_) between males and females. (**B**) Comparison of dose-adjusted estimated area under the daily concentration–time curve of daptomycin (eAUC_0–24h_/D) between males and females. (**C**) Comparison of plasma trough concentrations (C_min_) between males and females. (**D**) Comparison of dose-adjusted plasma trough concentrations (C_min_/D) between males and females. (**E**) Comparison of plasma peak concentrations (C_max_) between males and females. (**F**) Comparison of dose-adjusted plasma peak concentrations (C_max_/D) between males and females. (**G**) Comparison of drug clearance (CL) between males and females. * = *p* value < 0.01; ** = *p* value < 0.001; *** = *p* value < 0.0001.

We considered the main patients’ features which could impact daptomycin exposure. Out of 170 measurements, 95 were conducted for males and 75 for females. Females are overexposed to daptomycin compared with males as demonstrated by the main pharmacokinetics parameters, such as eAUC_0–24h_, trough and peak plasma concentrations, and drug clearance, and also when adjusted for the administered dose (Figure 5).

No statistical differences were observed between males’ and females’ CrCL values (*p* value = 0.12). The median lean body weight was 54.38 kg (females 48.87 kg, males 61.05 kg).

Due to different body composition between males and females, we performed an analysis to investigate the possible impact of the BMI and the estimated lean weight in females and males. A *t*-test showed statistically significant differences in estimated lean weight between males and females: *p* value < 0.001 and median values of 48.87 kg and 61.05 kg, respectively, for females and males. On the contrary, the same analysis considering BMI did not point out statistical differences (*p* value = 0.225) according to sex with median values of 28.32 kg/m^2^ for females and 26.93 kg/m^2^ for males.

**Figure 6:**
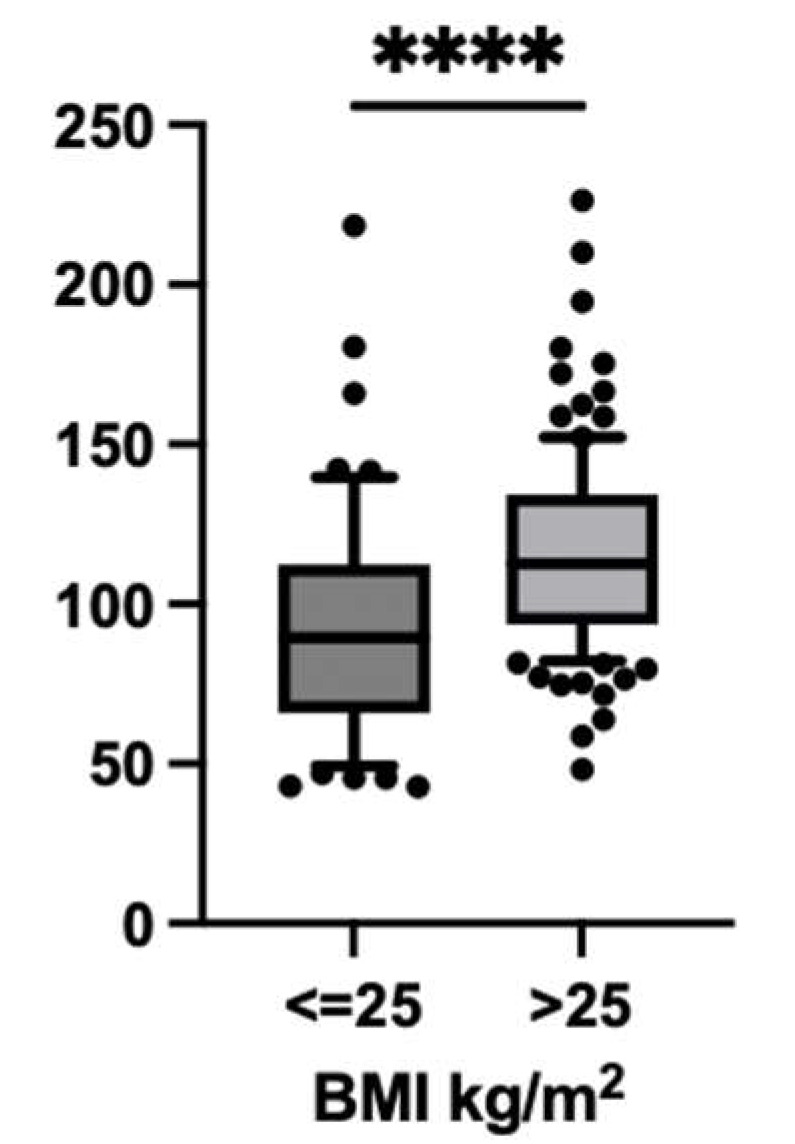
Comparison of dose-adjusted estimated area under the daily concentration–time curve of daptomycin (eAUC_0–24h_/D) between patients with normal weight (BMI ≤ 25 kg/m^2^) and overweight patients (BMI > 25 kg/m^2^). **** = *p* value < 0.00001.

No relevant correlation was found between lean weight and eAUC_0–24h_/D (R = −0.081, *p* value = 0.486), whereas a correlation was found between BMI and eAUC_0–24h_/D (R = 0.3559; *p* value = 0.001). Consistently, BMI influenced the exposure to daptomycin, as shown by comparing the median dose-adjusted eAUC_0–24h_ between patients with BMI ≤ 25 kg/m^2^ and with BMI > 25 kg/m^2^, which was 89.58 (IQR: 42.87–112.5) and 112.6 (IQR: 48.14–134.2), respectively (Figure 5 and Figure 6).

#### 3.3.2. Principal Component Analysis

Principal Component Analysis was used to relate differences in daptomycin clearance and plasma concentration according to gender.

**Figure 7:**
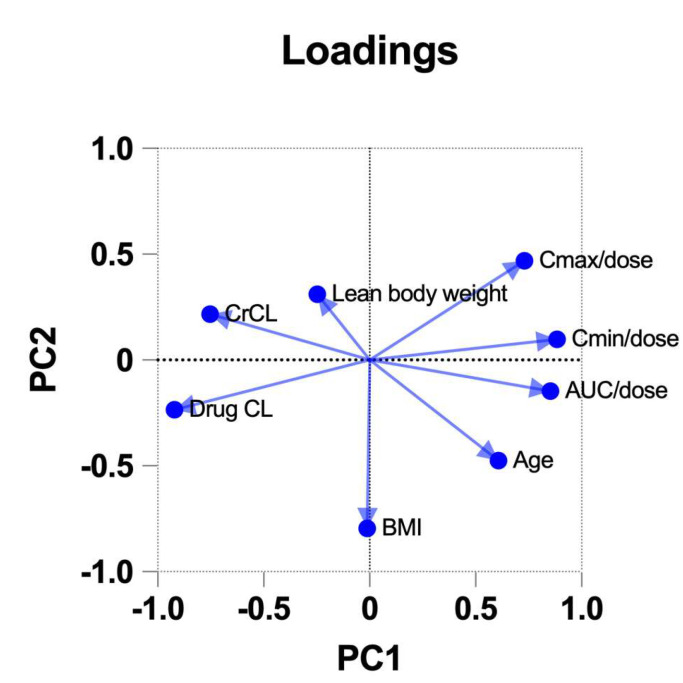
Vectors with loading values close to ±1 on the X-axis are strongly correlated with PC1, which represents the maximum amount of variance of data. The loading plot also illustrates the relationship between variables as described below.

The Principal Components are linear combinations of the continuous variables (CrCL, drug clearance, BMI, lean weight, age, AUC_0–24h_/dose, trough and peak concentration/dose). PC1 and PC2 were reported in the loading plot (Figure 7), where it can be observed that CrCL, drug clearance, AUC_0–24h_/dose, trough and peak concentration/dose are strongly correlated with PC1 (loadings values close to 1/−1), while BMI and, to a lesser degree, age are correlated with PC2.

Focusing on PC1, drug clearance and CrCL cluster together, which indicates that these variables are positively correlated, and the same occurs on the other side for AUC_0–24h_/dose and trough and peak concentration/dose (the higher the drug concentrations, the higher the drug exposure). On the other hand, variables with angles close to 180° are negatively correlated: the higher the drug clearance, the lower the drug exposure (AUC_0–24h_/trough and peak concentrations).

**Figure 8:**
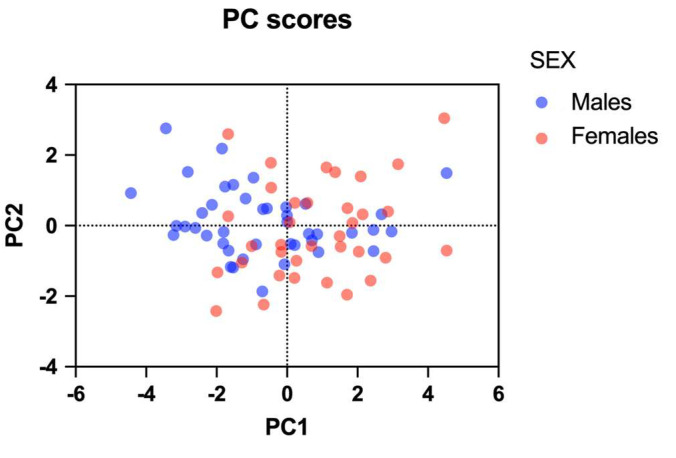
The score plot points out the tendency of clustering based on sex (males in blue, females in red) according to the vectors described in Figure 7. Males tend to cluster on the left side of the score plot (higher values of CrCL and drug clearance, lower values of plasma concentrations/dose and AUC_0–24h_/dose); females tend to cluster on the right side of the score plot (lower values of CrCL and drug clearance, higher values of plasma concentrations/dose and AUC_0–24h_/dose).

In the score plot (Figure 8), the two populations (Male vs. Female) tend to cluster as females are more likely to have lower CrCL and drug clearance values combined with a higher AUC_0–24h_, trough and peak/dose daptomycin concentrations than males.

#### 3.3.3. Safety and Efficacy

Patients were monitored for CPK and eosinophils during therapy. The data included 21 measurements of CPK levels both at basal time and during TDM. Of these, 14 patients showed CPK concentrations already >100 U/L at baseline, while an increase in CPK during therapy was observed in 6 patients. Only one patient reached high plasma CPK concentrations (1000 U/L), starting from a baseline of 325 U/L, and was characterized by an eAUC_0–24h_ of 1051 mg·h/L, with a plasma trough and peak concentration of 18.7 mg/L and 84.4 mg/L, respectively. Concomitant therapy with statins was reported only in six patients.

Data from 132 eosinophil count measurements at basal time and during TDM showed an increase in absolute eosinophil count at follow-up in 108 cases, with a mean variation in absolute eosinophil count of +138.3 eοsinophils/uL, ranging from −360 eοsinophils/uL to +820 eοsinophils/uL. At first TDM follow-up, five patients treated with daptomycin (7.8%) reported absolute eosinophil count > 500 eοsinophils/uL.

No other adverse events were reported.

As a surrogate of the efficacy marker, the CRP was evaluated during each TDM (data available for 155 measurements), with a median value of 28.99 mg/L.

The Mann–Whitney test showed statistical differences (*p* value = 0.029) in terms of CRP when an aggressive eAUC_0–24h_ (>939 mg·h/L) was achieved compared to patients reaching eAUC_0–24h_ < 939 mg·h/L, with median values of CRP, respectively, of 17.47 mg/L and 40.60 mg/L. No statistical differences were observed (*p* value = 0.889) in dose/kg between these two groups. Patients with an eAUC_0–24h_ < 939 and >939 mg·h/L showed statistical differences regarding eCrCL and BMI (p value, respectively, of 0.021 and 0.006). The median eCrCL values observed were 107.1 (IQR of 80.84–133.6 mL/min) and 90.43 mL/min (IQR of 66.48–124.5 mL/min), respectively, for the <939 and >939 mg·h/L group, while the median BMI values were 25.38 kg/m^2^ (IQR of 22.43–29.51 kg/m^2^) and 26.99 kg/m^2^ (IQR of 25.07–30.49 kg/m^2^).

## 4. Discussion

Daptomycin remains a valuable therapeutic option in various clinical settings, particularly given the increasing frequency of infections caused by pathogens with increased vancomycin MICs. This is due to the pharmacological properties of daptomycin, which are primarily related to its mechanism of action and its significant ability to penetrate tissues that are typically poorly reached by adequate concentrations of many common antibacterial agents. For this reason, daptomycin is also used, off-label, with high doses for BJIs. Due to significant challenges related to dose-dependent adverse events associated with daptomycin, administering the appropriate dose of this drug is particularly challenging [17,36]. When doses ranging from 8 to 10 mg/kg of daptomycin are used to achieve adequate therapeutic concentrations, there is a risk of exposing the patient to increased accumulation of daptomycin, potentially leading to toxicity. This complexity is further compounded by the characteristic significant intra- and inter-subject variability of daptomycin, which is difficult to predict based on standard clinical and laboratory parameters, such as body weight and renal function, as suggested by registration studies and the drug’s SmPC [14]. For this reason, TDM of daptomycin is employed in clinical practice. However, due to limitations such as the need for adequately trained personnel and organizational and economic constraints that prevent the implementation of daptomycin’s TDM in clinical practice, little is known in real-life settings about the PK/PD profile of this drug. Therefore, clinicians must manage the dosing of daptomycin on their own, risking inadequate drug concentrations, especially when administering high doses.

The analysis conducted at our University Hospital, one of the few institutions offering a daptomycin TDM service, sheds light on this clinical area and provides data that are useful for clinicians in the management of this drug. The PK/PD results, derived from routine clinical practice, and the PCA analyses confirm the significant impact of patient renal function on daily drug exposure. Furthermore, our data show that both BMI and female gender are significant factors modifying daily exposure to daptomycin to consider on top of already known parameters indicated in the pertinent SmPC [14]. Increased BMI and female gender are associated with increased exposure to daptomycin compared to individuals with a BMI ≤ 25 kg/m^2^ or male patients, even when administered at the same daily dose. In the subjects analyzed in this study, more than half of the patients (68.8%) did not receive adequate doses for the PK/PD efficacy and safety targets at the dosing suggested in the daptomycin SmPC. In 7.8% of cases, patients received subtherapeutic doses, while 61% had plasma concentrations associated with an increased risk of toxicity, which should suggest that the standard dose usually overexposes patients, and a lower dose could be sufficient for this clinical setting. Nevertheless, despite these concentrations, the available toxicity marker data showed no significant pharmacovigilance signals.

Focusing on the efficacy/safety balance of daptomycin, interestingly, in 20.1% of cases, patients’ 24 h exposure concentrations were associated with an increased risk of daptomycin toxicity, although trough concentrations were below the threshold of 24.3 mg/L, which is one of the most commonly used parameters to assess the risk of adverse events with daptomycin [15], as shown in Figure 2.

Despite all the evidence confirming the significant variability of an effective drug like daptomycin, its TDM is still not widely available to clinicians. Indeed, due to the aforementioned economic and organizational reasons, this service faces several barriers to its implementation in clinical practice. An accurate cost–benefit analysis of daptomycin therapy guided by TDM is not yet available due to limitations primarily related to the challenging assessment of measurable outcomes and to the different methods and organizational systems that the TDM service can offer to clinicians in daily practice. Additionally, clinical studies involving daptomycin use with TDM are highly heterogeneous, making it difficult to obtain generalizable data. However, while there are still challenges in identifying reference PK/PD targets to support its use in clinical practice and achieve clinically effective drug concentrations, in settings with limited financial and personnel resources that restrict plasma daptomycin measurement, TDM can be particularly useful for clinicians in assessing its toxicity risk. Indeed, the relationship between plasma daptomycin concentrations and the risk of toxicity has been extensively investigated. For this reason, a single blood sample for daptomycin TDM can be valuable for this purpose, especially in conditions that appear to be more associated with daptomycin overexposure, such as females and/or patients with high BMI, as also confirmed by the data presented in this study.

Unfortunately, the retrospective nature of this analysis, the limited number of patients studied, and the method of data collection do not allow for an adequate and reliable assessment of the PK/PD profile of daptomycin in the studied patients, nor its association with the incidence of its main side effects, particularly the most severe ones such as DIEP. Additionally, this study design is not able to measure the short- and long-term clinical efficacy of its dosing regimens, represented, for example, by the cure rate and time to cure in the short term, and by the relapse rate in the long term. However, this type of measurement would still be challenging to analyze even in the context of a prospective clinical trial, primarily due to the complexity of the clinical course of BJIs, the potential involvement of multiple pathogens, and the variables related to therapeutic options, which can introduce bias in attributing efficacy. The effectiveness of therapeutic treatments for these conditions is significantly influenced not only by the drugs used—which rarely involve monotherapy with a single antibacterial—but also by potential surgical interventions. The outcomes of such surgeries are typically operator-dependent, making them difficult to standardize and compare, as would be required in a clinical trial.

Overall, the studied patients had a good renal function (median eCrCL of 70.2 mL/min with IQR of 51.5–87.2 mL/min), with only two patients with a CrCL < 30 mL/min. However, in four patients (5.2%), the drug was administered every other day as recommended in the SmPC in view of the concentration-dependent activity of daptomycin. Nevertheless, estimation of the 48 h exposure during the dosing showed that the concentrations on the day without drug administration (eAUC_24–48h_) fell below the empirical therapeutic range (median of 339.8 mg·h/L; IQR: 290.5–396.6 mg·h/L). Since daily AUC is the PK/PD efficacy index in the main in vivo and clinical studies, alternate-day daptomycin administration may reduce the risk of drug accumulation but could also reduce the intensity of care required for optimal infection control and minimize the risk of developing resistance to daptomycin [37,38]. For this reason, a TDM approach could allow the administration of low daily doses of daptomycin compared to standard doses every 48 h.

This investigation has some limitations worth mentioning: the monocentric and retrospective nature of the study design, the limited inclusion of patients with impaired renal function, and the lack of microbiological and clinical outcomes assessment. Despite these limitations, these real-world data add new insights on PK/PD aspects about off-label use of high-dose daptomycin, specifically in patients diagnosed with BJIs, where little evidence exists. Due to the limitations, a prospective and preferably multicentric, phase IV study, also focusing on hard clinical endpoints and including patients with different renal functions, is required to confirm and generalize our results.

## 5. Conclusions

The use of daptomycin may represent a valid therapeutic option for BJIs. However, the variability of its PK profile makes this drug difficult to manage, as there is a risk of exposing patients to subtherapeutic or excessive doses. For this reason, further prospective studies with larger numbers of patients are needed to specifically investigate the PK/PD profile of daptomycin for BJIs. Finally, these data highlight how TDM for daptomycin can serve as a useful tool to personalize therapy for these infections, particularly considering the recent possibility of estimating daily drug exposure using even a single blood sample [18].

## Figures and Tables

**Table 1:** Patients’ characteristics at the baseline.

Variable	N (%) or Median (IQR)
Sex (Males/Females)	42/35 (54.5/45.5)
Age (years)	72 (60–77)
BMI ^1^ (kg/m^2^)	26.99 (23.68–40.04)
CrCL ^2^ (mL/min)	70.2 (51.5–87.2)
Initial daptomycin daily dose (mg/day)	800 (700–900)
Initial daptomycin daily dose per kg (mg/kg)	10 (9.3–10.4) Minimum: 4.6 Maximum: 12.7

^1^ Body Mass Index. ^2^ CrCL = estimated Creatinine Clearance according to the Cockroft–Gault formula, adjusted for the ideal weight.

## Data Availability

Data are available upon reasonable request from the corresponding author.
